# Supplementary material for: Bound Electron Enhanced Radiosensitisation of Nimorazole upon Charge Transfer
Source: Molecules. 2022 Jun 28;27(13):4134. doi: 10.3390/molecules27134134 (PMC9268075; doi:10.3390/molecules27134134)
Supplement: Supplementary file 1 [file molecules-27-04134-s001.zip › molecules-1776204-supplementary.pdf]

Supplementary Materials

Bound electron enhanced radiosensitisation of nimorazole upon charge transfer

S. Kumar [1], I. B. Chouikha [2], B. Kerkeni [2,3*], G. García [4], P. Limão-Vieira [1*]

1 Atomic and Molecular Collisions Laboratory, CEFITEC, Department of Physics, Universidade NOVA de Lisboa, Campus de Caparica, 2829-516 Caparica, Portugal; s.kumar@campus.fct.unl.pt (S.K.); plimaovieira@fct.unl.pt (P.L.-V.)

2 Département de Physique, LPMC, Faculté des Sciences de Tunis, Université de Tunis el Manar, Tunis 2092, Tunisia; islem.benchouikha@fst.utm.tn (IBC); boutheina.kerkeni@obspm.fr (B.K.)

3 ISAMM, Université de la Manouba, La Manouba 2010 Tunisia; boutheina.kerkeni@obspm.fr (B.K.)

4 Instituto de Física Fundamental, Consejo Superior de Investigaciones Científicas, Serrano 113-bis, 28006 Madrid, Spain; g.garcia@csic.es

* Correspondence: plimaovieira@fct.unl.pt (P.L.-V.); boutheina.kerkeni@obspm.fr (B.K.)

*Correspondence: boutheina.kerkeni@obspm.fr; plimaovieira@fct.unl.pt

TDDFT/ M06-2X/6-311++g(d,p) computational model was used in electronic structure calculations. All electrons have been considered explicitly for carbon, oxygen, nitrogen, hydrogen and potassium atoms with the 6-311++g(d,p) basis set

Figure caption

Figure S1: Fully optimised geometry of nimorazole at the M06-2X/6-311++g(d,p) level of theory. Fully optimized molecular structure of the K + nimorazole collisional system K–O ≈ 5.1 Å. K: yellow, O: red, C: grey, N: light blue, and H: white. Cartesian coordinates (in Å).

Figure S2: Energy (in eV) and shape of a selection of the molecular orbitals (TDDFT/ M06-2X/6-311++g(d,p)) for K + NIMO (K: purple, C: grey, N: blue, O: red, and H: white). The straight lines between the K atom and the –NO_2 end in the nitroimidazole ring are just to indicate the spatial mutual position.

Figure S3: Energy (in eV) and shape of a selection of the molecular orbitals (M06-2X/6-311++g(d,p)) for NIMO (C: grey, N: blue, O: red, and H: white).

Figure S1: Fully optimised geometry of nimorazole at the M06-2X/6-311++g(d,p) level of theory. Fully optimized molecular structure of the K + nimorazole collisional system K–O ≈ 5.1 Å. K: yellow, O: red, C: grey, N: light blue, and H: white. Cartesian coordinates (in Å).

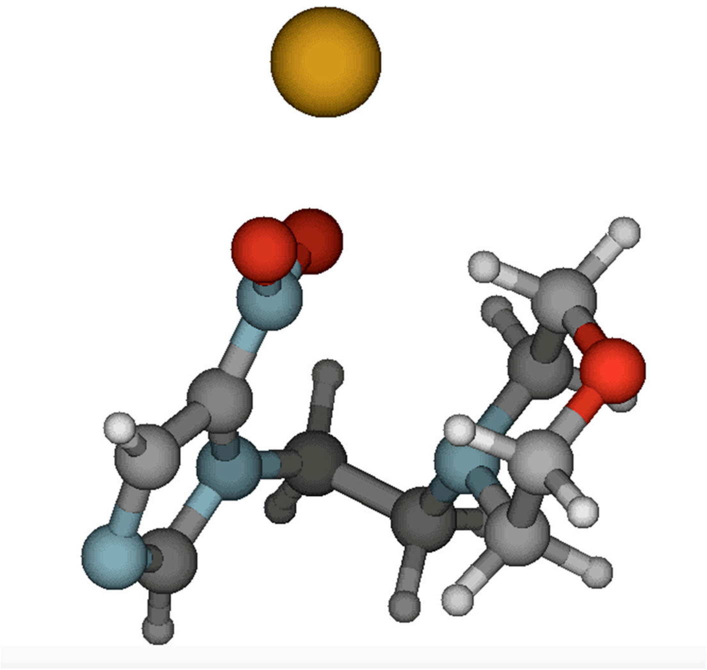

Cartesian coordinates (in Å).

C	-1.420369	0.935288	-0.002623
C	-2.173875	1.301569	1.093023
C	-2.763699	-0.681986	0.585502
N	-1.812232	-0.343711	-0.339432
H	-2.145992	2.245774	1.609137
H	-3.241564	-1.651108	0.562053
N	-0.481321	1.677568	-0.687116
O	-0.095453	2.803273	-0.171942
O	-0.108712	1.352728	-1.894973
C	-1.127140	-1.314816	-1.183171
H	-1.863916	-2.060121	-1.492879
H	-0.752059	-0.795845	-2.061864
C	0.008225	-1.982200	-0.400485
H	0.468590	-2.770692	-1.019343
H	-0.419992	-2.467095	0.481591
N	0.970923	-0.991773	0.034944
O	3.361762	0.290946	0.792860
C	2.007597	-0.717105	-0.945513
H	1.537980	-0.493391	-1.905521
H	2.686518	-1.582196	-1.061822
C	2.803816	0.495525	-0.490399
H	2.127998	1.362282	-0.463349
H	3.640056	0.686577	-1.166570
C	2.340919	0.030164	1.742421
H	2.837580	-0.109915	2.702912

H	1.660440	0.890794	1.799458
C	1.541132	-1.205151	1.354725
H	2.199143	-2.092655	1.383743
H	0.729244	-1.347031	2.073211
N	-3.010278	0.275255	1.441875
K	0.993434	3.583190	-2.269275

Figure S2: Energy (in eV) and shape of a selection of the molecular orbitals (VTZ/6-311G) for K + NIMO (K: purple, C: grey, N: blue, O: red, and H: white). The straight lines between the K atom and the $–NO_2$ end in the nitroimidazole ring are just to indicate the spatial mutual position.

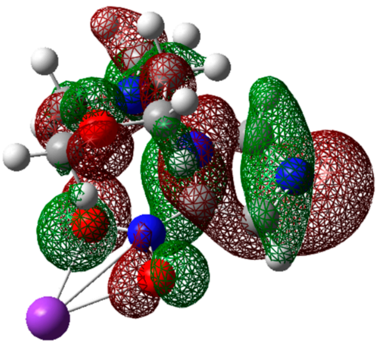	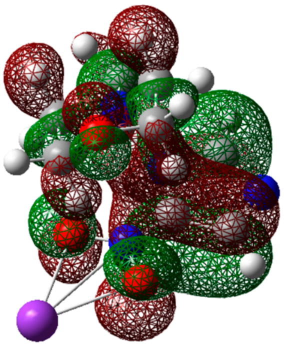
HOMO-3 (-8.57)	HOMO-2 (-7.86)
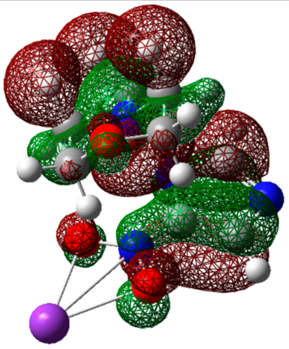	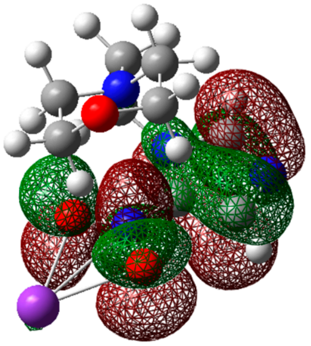
HOMO-1 (-7.22)	HOMO (-5.02)
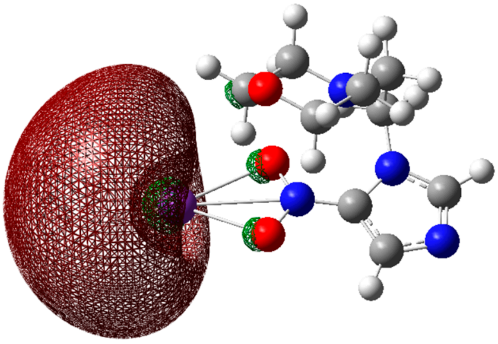	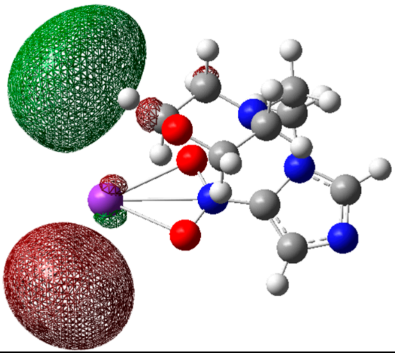
LUMO (-1.25)	LUMO+1 (-0.33)
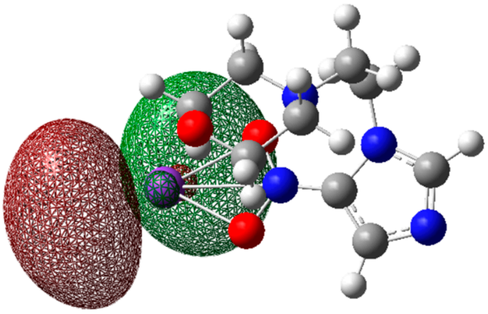	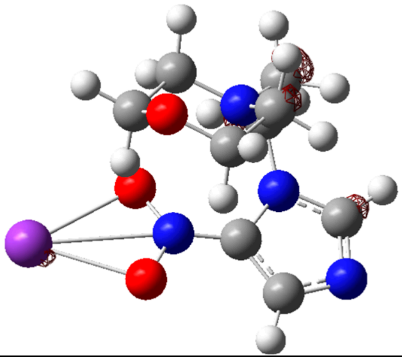
LUMO+2 (-0.27)	LUMO+3 (-0.07)

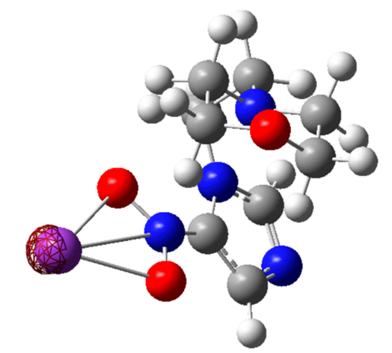	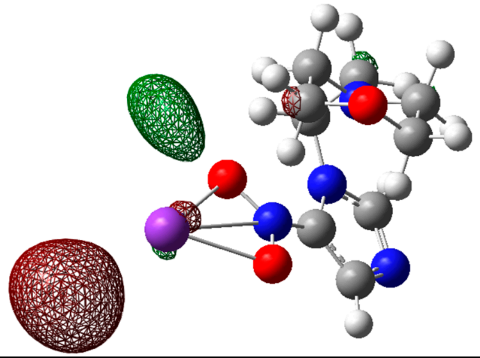
LUMO+4 (0.01)	LUMO+5 (0.23)
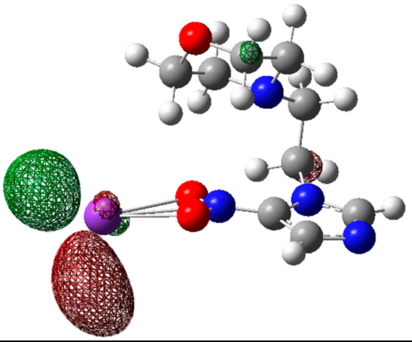	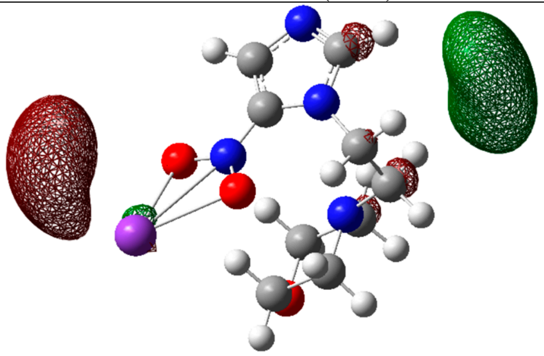
LUMO+6 (0.26)	LUMO+7 (0.38)
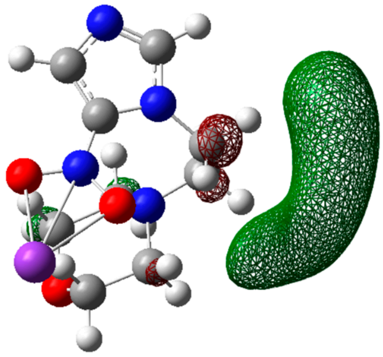	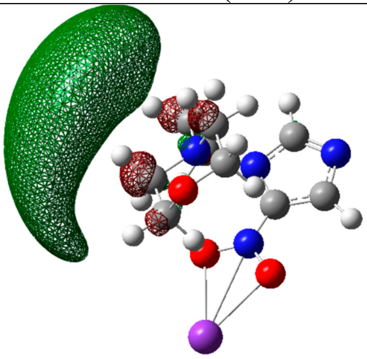
LUMO+8 (0.54)	LUMO+9 (0.60)
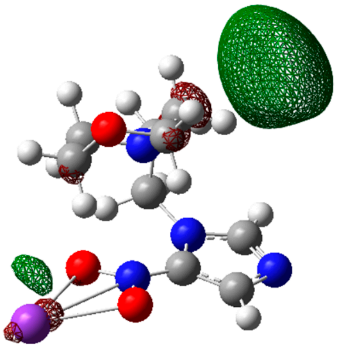	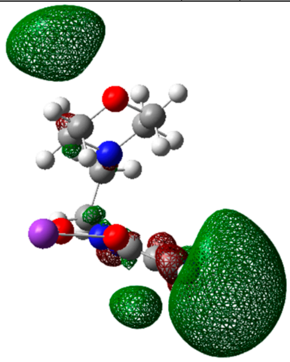
LUMO+10 (0.76)	LUMO+15 (1.32)

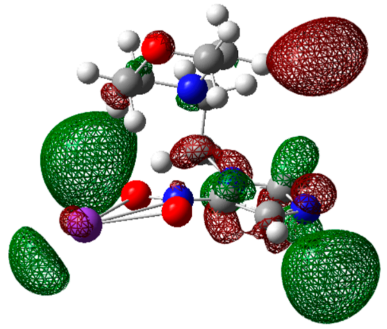	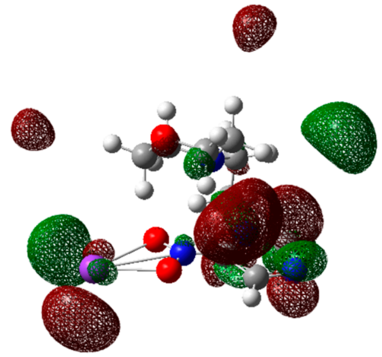
LUMO+20 (1.84)	LUMO+25 (2.39)
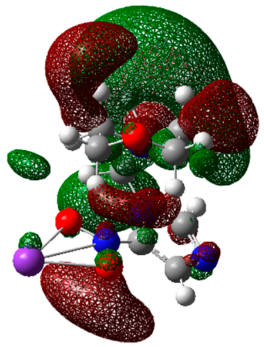	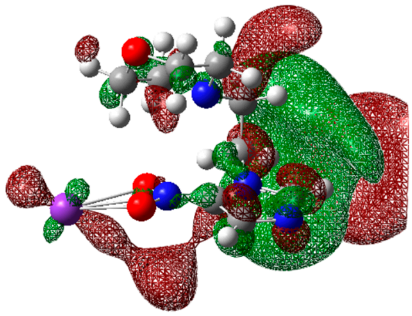
LUMO+30 (2.93)	LUMO+35 (3.51)
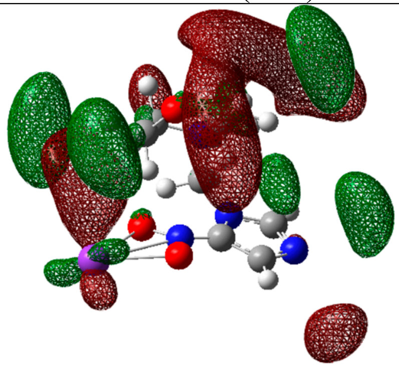	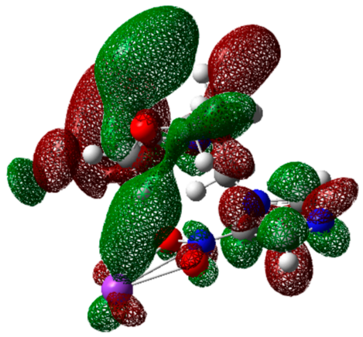
LUMO+40 (3.94)	LUMO+45 (4.38)
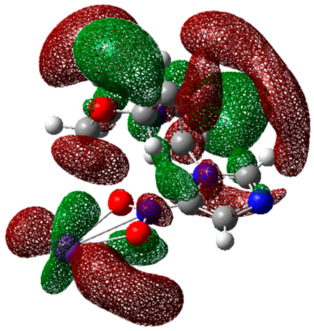	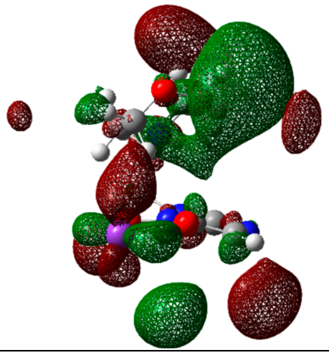
LUMO+50 (4.81)	LUMO+55 (5.11)

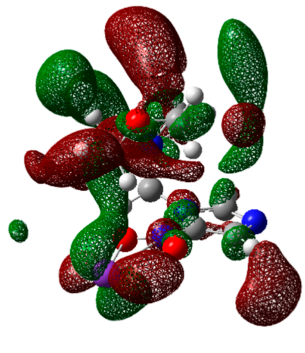	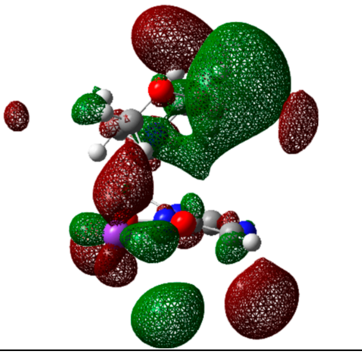
LUMO+56 (5.20)	LUMO+60 (5.69)
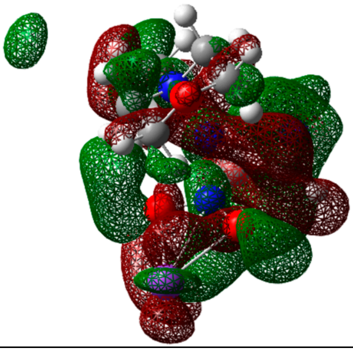	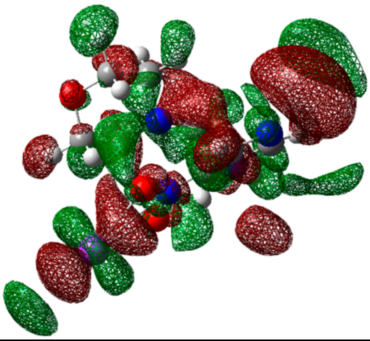
LUMO+70 (7.01)	LUMO+80 (8.28)
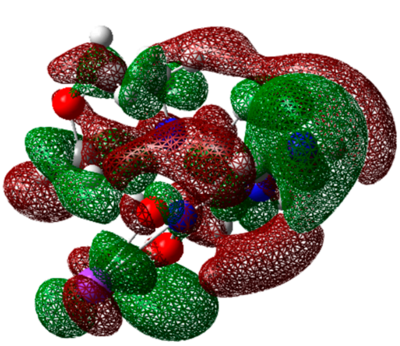	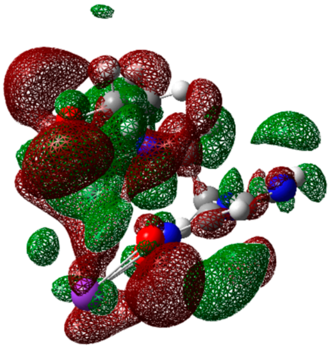
LUMO+90 (9.17)	LUMO+100 (10.01)

Figure S3: Energy (in eV) and shape of a selection of the molecular orbitals (M06-2X/6-311++g(d,p)) for NIMO (C: grey, N: blue, O: red, and H: white).

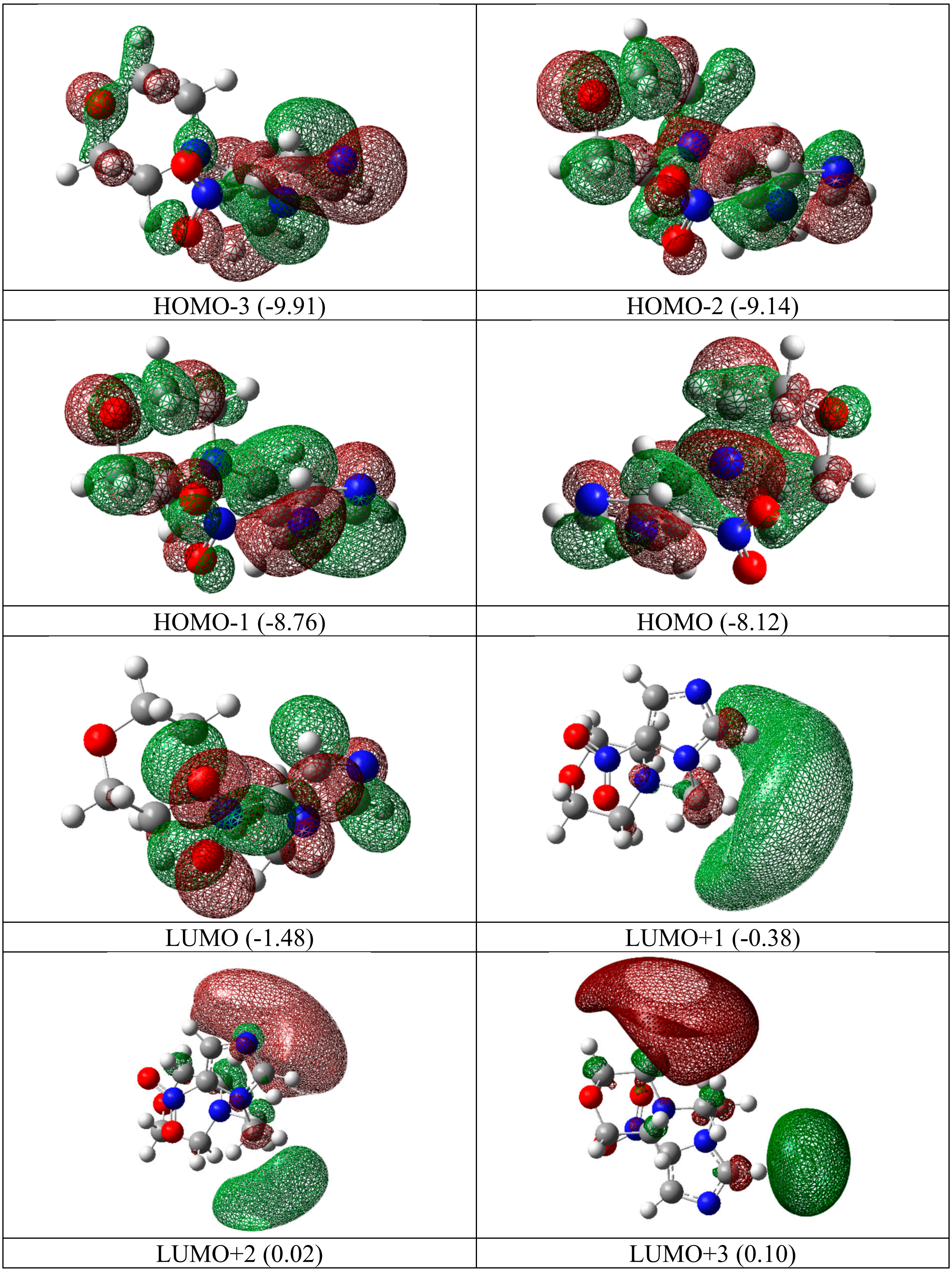

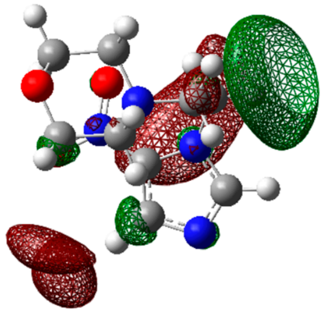	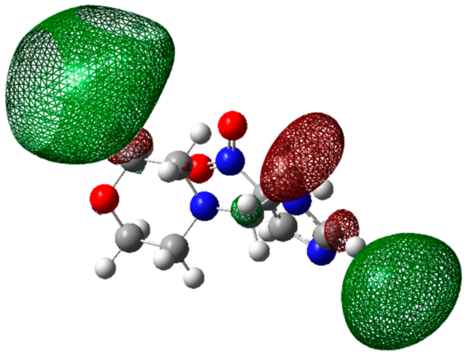
LUMO+4 (0.22)	LUMO+5 (0.56)
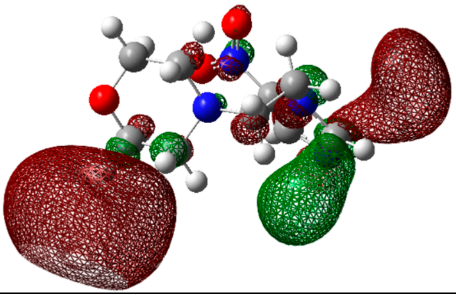	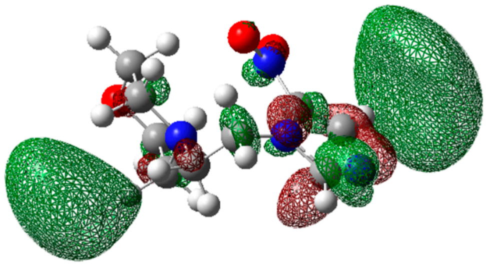
LUMO+6 (0.61)	LUMO+7 (0.63)
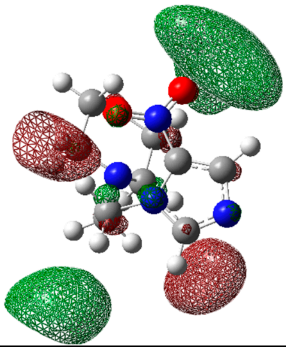	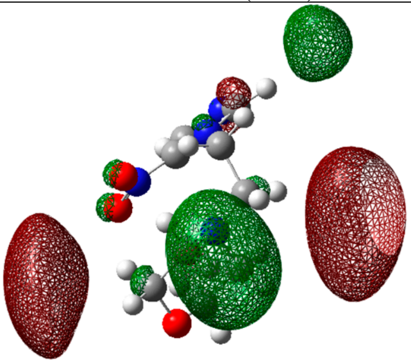
LUMO+8 (0.75)	LUMO+9 (1.00)
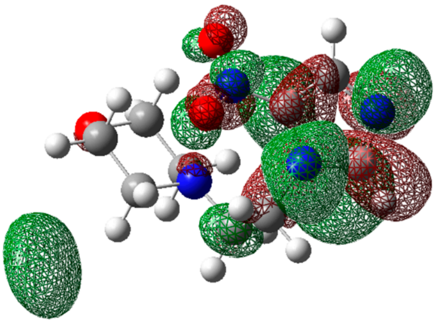	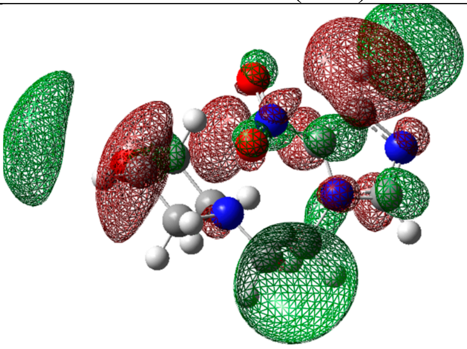
LUMO+10 (1.16)	LUMO+15 (1.66)

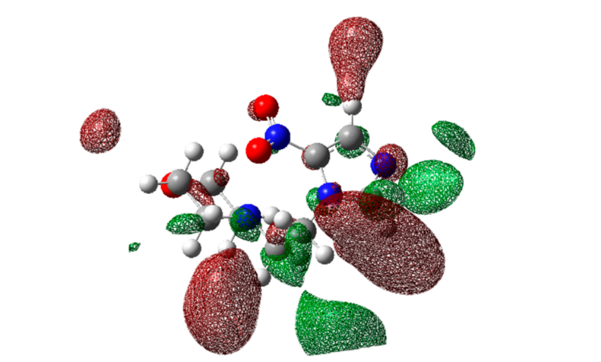	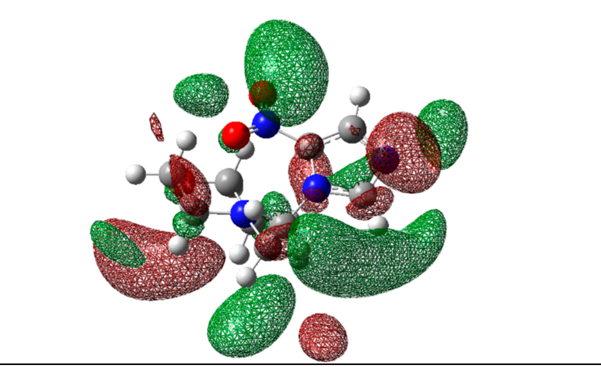
LUMO+20 (2.40)	LUMO+25 (3.05)
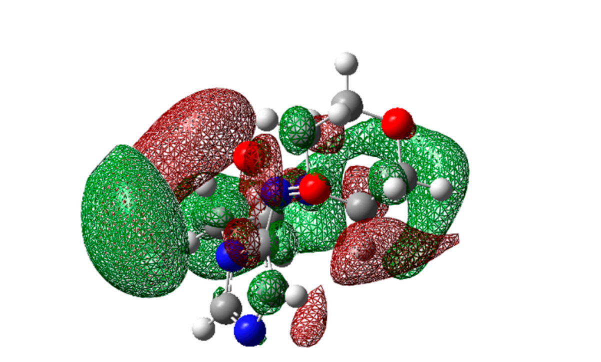	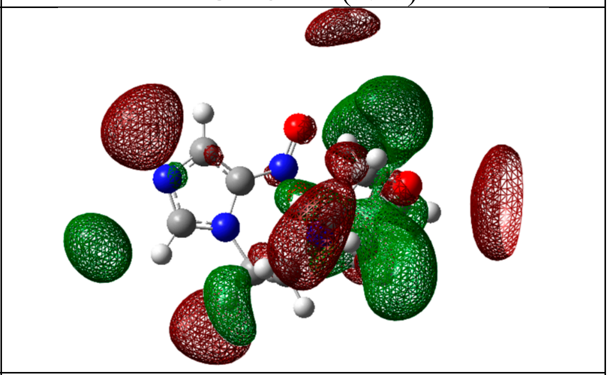
LUMO+30 (3.54)	LUMO+40 (4.28)
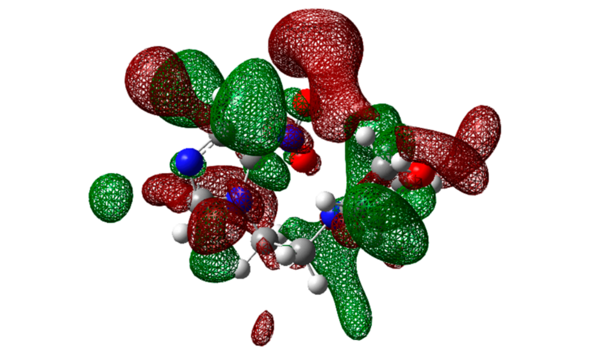	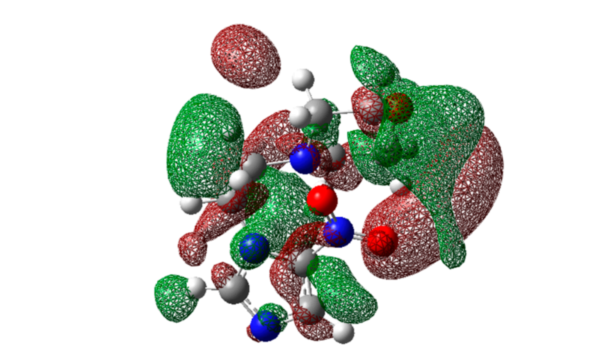
LUMO+50 (5.28)	LUMO+56 (5.41)
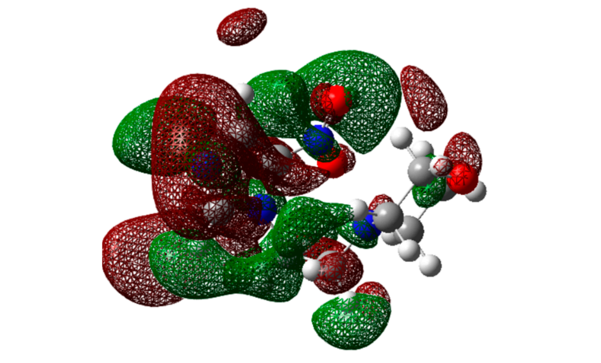	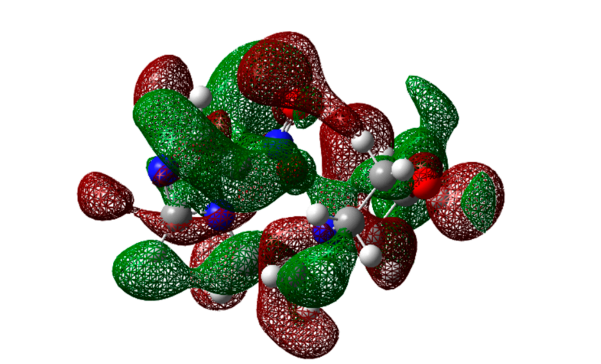
LUMO+60 (6.68)	LUMO+70 (7.80)

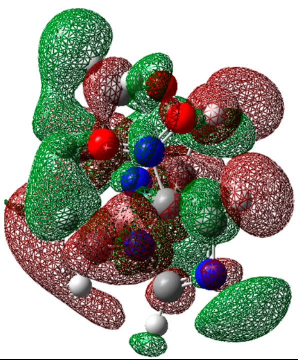	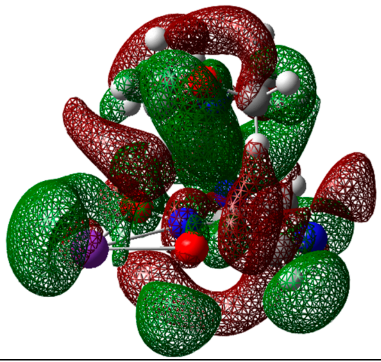
LUMO+80 (8.86)	LUMO+90 (10.03)
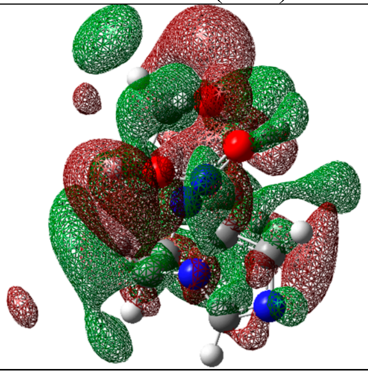	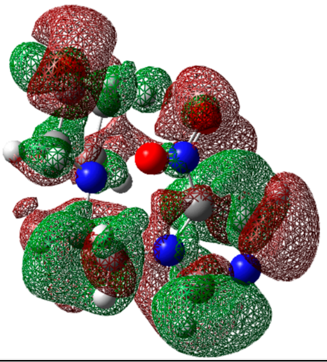
LUMO+100 (11.53)	LUMO+120 (17.30)
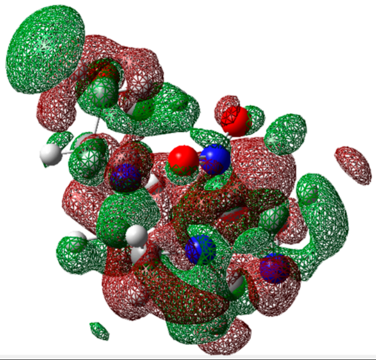	
LUMO+140 (21.5)	
